# A patient from Mexico with vaping-associated lung injury, seizures and renal failure

**DOI:** 10.18332/tid/114316

**Published:** 2019-12-11

**Authors:** Alejandro E. Macias, Francisco J. Garcia, Sonia G. Saldana

**Affiliations:** 1 Department of Medicine, University of Guanajuato, Leon, Mexico; 2 Department of Intensive Care, Medica Campestre Hospital, Leon, Mexico

**Keywords:** e-cigarette, vaping, disease

**Dear Editor,**

For vaping, a liquid is heated to create an inhalable aerosol. ‘E-liquids’ may contain nicotine, glycerol, nitrosamines, aldehydes, metals, organic compounds, phenolic compounds, polycyclic hydrocarbons, flavorings, alkaloids, and drugs; some of these may cause lung damage, particularly if adulterated^1-3^. In the US, the FDA has issued also an alert on vaping-associated seizures; the agency has received 127 reports of seizures that occurred between 2010 and 2019^4^. An outbreak of lung injuries associated with vaping has been reported with 2172 acute cases and 42 deaths, in the US. In about 86% of the cases, people reported use of products containing tetrahydrocannabinol (THC), many from street vendors^3,5^. Analyses of bronchoalveolar lavage fluid samples of patients with vaping-associated lung injuries has identified vitamin E acetate, an additive in some THC-containing products^3^.

**Figure 1:**
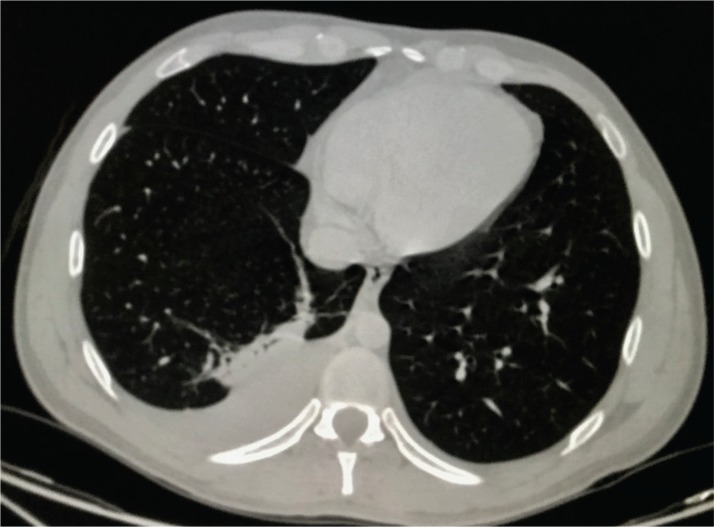
A chest tomography showing a right-side effusion together with a posterior-basal infiltrate in a patient using E-cigarettes

We present here a case in Mexico of a previously healthy 31-year-old male with seizures and lung damage that meets the CDC definition for probable vapingassociated lung injury^5^. The patient used daily e-cigarettes, containing nicotine and THC, which he acquired online (in Mexico, vaping products are illegal). On 8 September 2018, his father found him unconscious; while being transferred to the hospital he suffered two generalized tonic-clonic seizures. At the emergency department, he was stuporous and required intubation and mechanical ventilation due to acute respiratory distress syndrome (Kirby index, 90 mmHg). His blood tests reported leukocytosis (21000/uL with predominance of lymphocytes 11750/uL, eosinophiles 430/uL, neutrophiles 6620/uL), aminotransferases mildly elevated (AST 45.7 U/L, ALT 102.4 U/L), serum glucose was 272 mg/dL, and creatinine 1.3 mg/dL. Arterial blood gas showed mixed high anion gap acidosis. The spun urine specimen showed a countless amount of erythrocytes. The lumbar tap found normal fluid and the head CT was normal. The chest x-ray showed opacity at the right lung base; the CT showed a right pleural effusion and a posterior-basal pulmonary infiltrate (Figure 1). He received anticonvulsive drugs, two doses of systemic steroids for the first 48 hours (equivalent to 50 and 25 mg of prednisone), and antibiotics, although the clinicians did not believe that infection was the sole cause of the lung injury. After 72 hours, his serum creatinine reached 7 mg/dL. During the following days he did not suffer from additional seizures, his respiratory condition improved, and his renal failure resolved with hydration only. He was extubated on the fifth hospitalization day. He is currently healthy.

The patient had suffered from seizures, renal failure, and lung injury temporarily associated with vaping. There may have been many similar but not reported cases outside US. It is appropriate that the health alert effective in the US be observed globally and that particular care is taken to avoid the use of substances acquired in informal commerce.
